# Influence of Adding Carbonaceous Fuels to Ionic Liquids on Propellant Properties

**DOI:** 10.1021/acsomega.2c04386

**Published:** 2022-11-26

**Authors:** Xuyao Gao, Zhongquan Gao, Yonghua Tan, Pengfei Chen, Zenghui Du, Yutong Li

**Affiliations:** † State Key Laboratory of Multiphase Flows in Power Engineering, Xi’an Jiaotong University, Xi’an710049, People’s Republic of China; ‡ Xi’an Aerospace Propulsion Institute, China Aerospace Science and Technology Corporation, Xi’an710199, People’s Republic of China

## Abstract

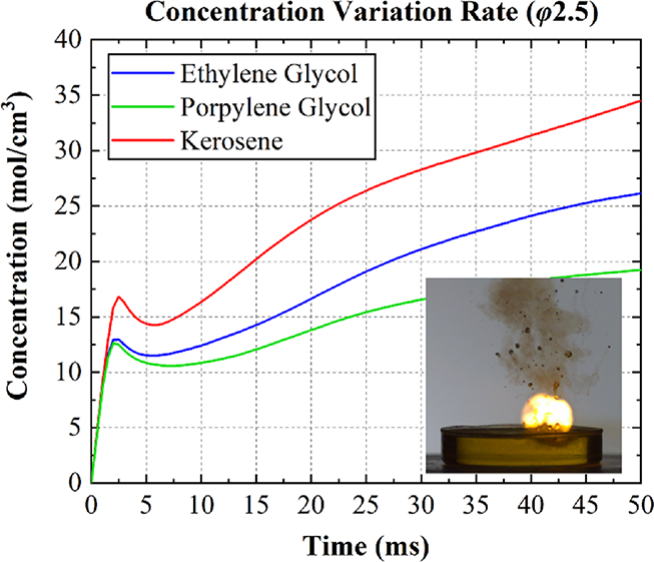

To make ionic liquids (ILs) accessible and economical, ethylene glycol was mixed in 1-ethyl-3-methylimidazolium-dicyanamide ([EMIm]DCA) to obtain droplets that could experimentally collide white fuming nitric acid. To investigate the ignition delay (ID) time theoretically in terms of hydrodynamics, alcohol fuels and kerosene were used as combustibles, while the intermiscibility between them and nitric acid (HNO_3_) was calculated using the ternary phase-field method alongside finite element analysis. The specific impulses of blend fuels were calculated by a thermodynamic method and compared to ILs. When the droplet was ethylene glycol/[EMIm]DCA with a 2.1 mm diameter and a 1.69 m/s colliding velocity, the ID time was the shortest. Kerosene was not an applicable additive for [EMIm]DCA owing to its lower intermiscibility with ILs and HNO_3_ than alcohol fuels; alcohol fuels, however, were appropriate. The concentration of ethylene glycol in the oxidizer pool increased faster than the concentration of propylene glycol, triggering more rapid hypergolic ignition in the first 50 ms. The protocols regarding the hypergolic ignition conditions were verified, i.e., the size of the droplet had to be minute when the colliding velocity was as fast as possible; this was carefully calculated using ethylene glycol. According to thermodynamic calculations, the addition of alcohol fuels can improve the specific impulse of fuels, with ethylene glycol performing the best. The feasibility of adding alcohol fuels to ILs was confirmed via experiments and thermodynamic computations, with the simulation results providing some guidance on selecting the experimental or engineering conditions or both.

## 1 Introduction

Bipropellants are widely applied in various areas, including liquid rocket engines, space engines, and guided missiles.^1^ Their application has several benefits, e.g., when used in space engines, attitude and orbit control can be implemented with the anticipative property of the engine being operated in pulse mode with a low thrust. Moreover, the wide range of thrust and higher specific impulses than solid propellant rockets render it important in rocket engines.

The use of hydrazine and hydrazine derivatives as traditional propellants has long been criticized for their toxicity, high volatility, and sensitivity to diabatic compression, which can result in operational difficulties.^2^ In 2008, the first protocol involving the use of dicyanamide (DCA)-based ionic liquids (ILs) as a propellant (rather than hydrazine) was proposed.^3^ However, given the growing concerns about ILs, considerable efforts have been made to establish more environmentally friendly and nontoxic hypergolic ILs.

Ignition occurs when DCA-based ILs react vigorously with fuming nitric acid (FNA). The resultant reaction products have been identified using Fourier transform infrared spectroscopy.^4^ Specifically, Li et al. conducted drop tests in which two ILs, i.e., [EMIm]DCA and 1-butyl-3-methylimidazolium ([BMIm]DCA), were mixed with three oxidizers (white fuming nitric acid [WFNA], red fuming nitric acid [RFNA], and nitrogen tetroxide [NTO]).^5^ Elsewhere, as a mature IL, [EMIm]DCA was investigated in terms of vapor pressure, thermal stability, specific heat, and ion fragmentation.^6−8^ Contrarily, WFNA, DCA-based ILs, do not react with either NTO or RFNA, which is essentially nitric acid (HNO_3_) with added NTO.

The ignition delay (ID) time is one of the most important criteria of IL performance, provided that hypergolic ignition occurs; the components and structure of ILs impact both the ID time and the ignition process,^9^ whereas in macroscopic terms, the viscosity of ILs and the experimental method (e.g., the velocity of contact, ambient temperature, and size of the droplet) are also influencing factors.^10−12^ In addition, specific impulse is also an important performance parameter for rockets. Except for the structural design of rockets, the choice of propellant is also a factor in determining the specific impulse.^13^ Specific impulse can be increased by decreasing water content in an oxidizer, or increasing heat of formation and energy-density of fuel.^14,15^ [EMIm]DCA is a less-than-spectacular fuel^5^ with respect to specific impulse compared with RP-1 and hydrazine derivatives.

In this study, [EMIm]DCA was selected as the IL and WFNA as an oxidizer, and the IL viscosity and the experimental methods were varied accordingly. Alcohol fuels were used as the combustible liquid and were mixed in the [EMIm]DCA to improve fuel economy. In addition to observing any emerging phenomena, a computational fluid dynamics (CFD) model was created to calculate the rate at which one liquid phase dissolved into another. The results of the simulation experiments provide a reference for subsequent experimental design and predictions. At last, the specific impulse calculation of the blended fuels can evaluate whether the alcohol fuel is a favorable additive.

## 2 Materials and Methods

### 2.1 Experimental Methods

**Figure 1:**
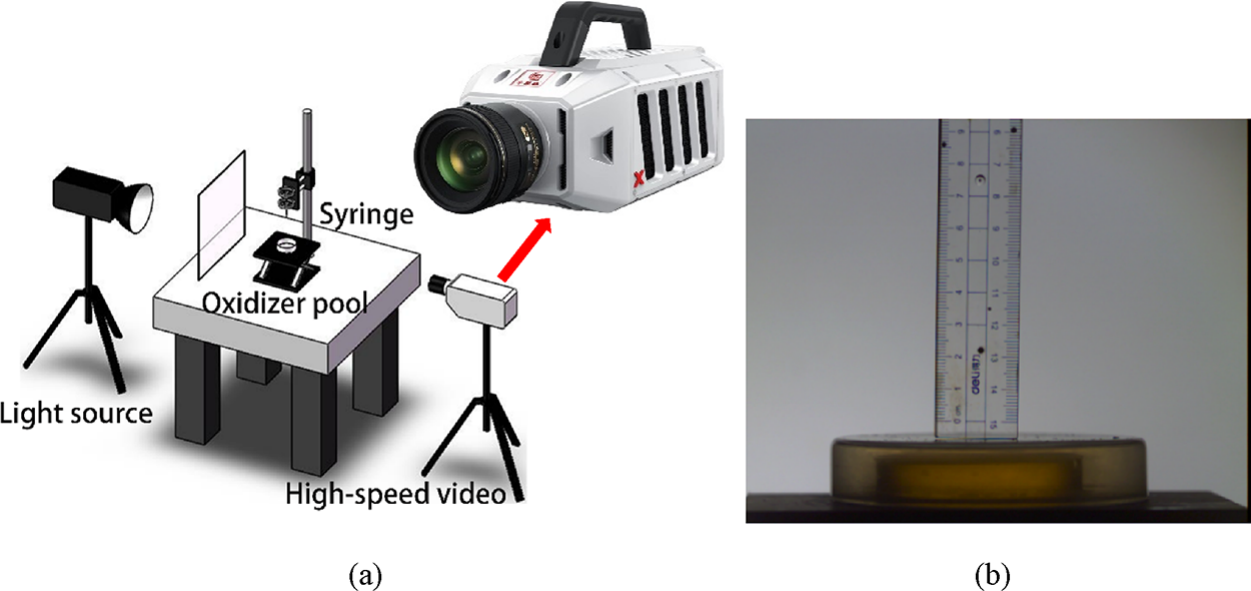
(a) Experimental setup; (b) calibration process.

A drop test involving a single droplet collision was conducted, and to identify an optimized solution, a variable-control approach was adopted. The experimental setup is shown in Figure 1a. Here, a syringe with the capacity for generating different-sized droplets was used as a droplet generator. The droplet goes into freefall when it leaves the syringe and collides with the oxidizer in the pool at a specific velocity. All these devices were supposed to remain in place so that the relative distances were constant, and then the gauge was calibrated by pixels. (Figure 1b) The height of the syringe was adjustable to provide different velocities, whereas a light source provided extra-strong light to enable the high-speed camera (Revealer X113) to shoot at 5000 frames per second.

#### 2.1.1 Variables

As noted, [EMIm]DCA was selected as the IL and three variables were selected for the experiment, i.e., collision velocity, droplet size, and fuel content, with the collision velocity set to 1.34, 1.69, and 1.9 m/s, based on the height of the syringe from the pool; the droplet size was set to 2.1, 2.5, and 2.9 mm in diameter. These two sets of data were obtained via the calibration calculation process shown in Figure 1b. Finally, different molar ratios were introduced to measure the content of the fuel, with the ratios of the IL set to 10, 20, and 30%, respectively. Every variable has three values, and there are three variables. Totally, 27 groups of experiments were processed. Each group was repeated three times, and the results were averaged.

#### 2.1.2 Fuel

The preparation cost of ILs is higher compared with traditional fuels; this is one reason that prohibits the widespread adoption of ILs in engineering contexts. In this study, alcohol fuels were used as combustibles and mixed into the [EMIm]DCA to improve fuel economy. Ethylene glycol and propylene glycol are widely used as combustible chemicals in many fields and are immiscible with [EMIm]DCA in any given proportion. The ethylene glycol and [EMIm]DCA mixture was labeled “EE,” whereas the propylene glycol and [EMIm]DCA mixture was labeled “PE,” as shown in Table 1. The calculation formula for the ideal liquid mixing viscosity is as follows:
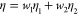
1where *w* is the mass fraction and η is the dynamic viscosity. The calculated results for the mixture viscosity are shown in Table 1. The slight differences between the densities of the fuels could be neglected and were not calculated in this study.

**Table 1:** Fuel Mixtures that Were Used in This Study

	content of ethylene glycol	viscosity (mPa·s)		content of propylene glycol	viscosity (mPa·s)
EE1	10%	16.88	PE1	10%	19.64
EE2	20%	17.61	PE2	20%	22.67
EE3	30%	18.23	PE3	30%	25.32

### 2.2 Numerical Model

#### 2.2.1 Governing Equations

The field of CFDs presents an emerging cross-discipline that integrates fluid mechanics and computer science. It uses a calculation method and rapid calculation capacity of a computer to obtain the approximate solution for a fluid control equation. In this paper, the ternary phase-field (TPF) method was adopted to simulate the immiscibility of the two liquids used in this study in the air.^16^ To describe the hydrodynamics of the mixture, the Cahn–Hilliard and the Navier–Stokes equations for incompressible flows were combined. In the phase-field interface, the two-phase flow dynamics were governed by the Cahn–Hilliard equation, which is often used to describe the evolution of phase boundaries in phase-field models for multiphase fluids,^17^ with the equation tracking a diffuse interface separating the immiscible phases. After generalizing the diphasic Cahn–Hilliard model noted above for the simulation of three immiscible component flows without phase changes, the final Cahn–Hilliard equations were obtained as follows:
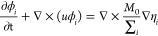
2
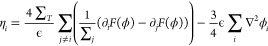
3where ε is the interface thickness control parameter, φ denotes a different phase (the default setting in COMSOL lets A stands for liquid pool, B stands for droplet, and C stands for air), η_*i*_ is the phase-field help variable, and *M*_0_ is a diffusion coefficient, which may depend on a smooth function known as the “mobility”.^18^

The Navier–Stokes equations describe the transport of mass and the momentum of fluids with a constant density^19−21^ and can be described as follows:
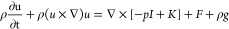
4
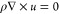
5
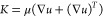
6where *u* is the velocity, *p* is the pressure, *T* is the temperature, *g* is the acceleration of gravity, and *F* is the bulk free energy. The mass transfer in the reactor domain is given by the transient convection and diffusion equation as follows:
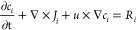
7
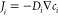
8where *D_i_* (m^2^/s) denotes the diffusion coefficient, *R_i_* (mol/[m^3^·s]) denotes the reaction term, and *c* refers to the concentration of one liquid phase (droplet of fuel) in another liquid phase (HNO_3_).

The multiphysics coupling feature determines the density and viscosity of the mixture to enable varying it smoothly over the interface by allowing the following:
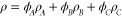
9
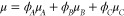
10
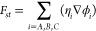
11where *F_st_* is the surface tension force.

#### 2.2.2 Simulation Conditions

The difference between specific mixture fuels can influence the numerical results. A practical protocol for clearly investigating the fluid dynamics involved magnifying this difference by only selecting alcohol fuels and kerosene as the liquid droplets in the simulation. The considered physical properties (at 16 °C) are shown in Table 2.

**Table 2:** Considered Physical Properties

	HNO_3_	(CH_2_OH)_2_	C_3_H_8_O_2_	kerosene
ρ (density) (g/cm^3^)	1.41	1.113	1.036	0.84
μ (viscosity) (mPa·s)	0.89	25.66	56	8
σ (surface tension) (mN/m)	58.6	46.49	38	30

**Figure 2:**
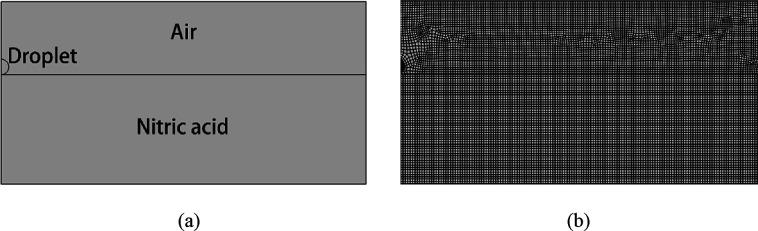
(a) Ternary phase-field conditions and (b) the meshing used in the model.

Working under the premise of two-dimensional axisymmetry, the air field was a 50 × 10 mm rectangle, the liquid field (liquid pool) was a 50 × 15 mm rectangle, and the other liquid field (fuel droplet) was a semicircle with varying diameters (2.1, 2.5, and 2.9 mm) under various simulation conditions. In accordance with the actual conditions, the initial collision velocities were set to 1.34, 1.69, and 1.9 m/s in the numerical model. Additionally, to perform the finite element grid division method, quadrilateral and triangular partitions were developed in the model (Figure 2).

### 2.3 Calculation of Specific Impulse

In the condition of uncertain type of rocket engine, equilibrium flow and frozen flow can be calculated by the infinite-area chamber model using Chemical Equilibrium Applications (CEA) code developed at NASA.^5^ In this model, the initial temperature (*T*_0_) and pressure (*P*_0_) were 298.15 K and 20 bar, respectively. The nozzle area expansion ratios were set as 30, 50, 70, and 90. According to the definition of blend fuels above, amounts of substance are shown in Table 3. The gradient of oxidant-to-fuel ratios were the same with every type of fuels to ensure a horizontal comparison.

**Table 3:** Fuels Content when Calculating Specific Impulse

		content of ethylene glycol (mol)	content of propylene glycol (mol)	content of ILs (mol)		HNO_3_ (mol)
fuels	ILs_1	0	0	11	oxidizers	
	ILs_2	0	0	12		60
	ILs_3	0	0	13		
	EE1	1	0	10		
	EE2	2	0	10		60
	EE3	3	0	10		
	PE1	0	1	10		
	PE2	0	2	10		60
	PE3	0	3	10		

## 3 Results

### 3.1 Experimental Results

**Figure 3:**
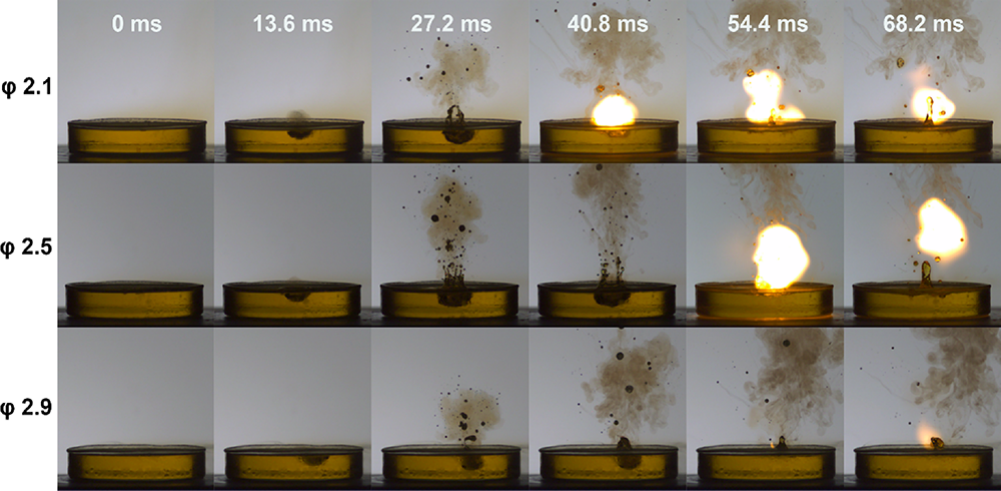
Recording of the ignition process.

**Figure 4:**
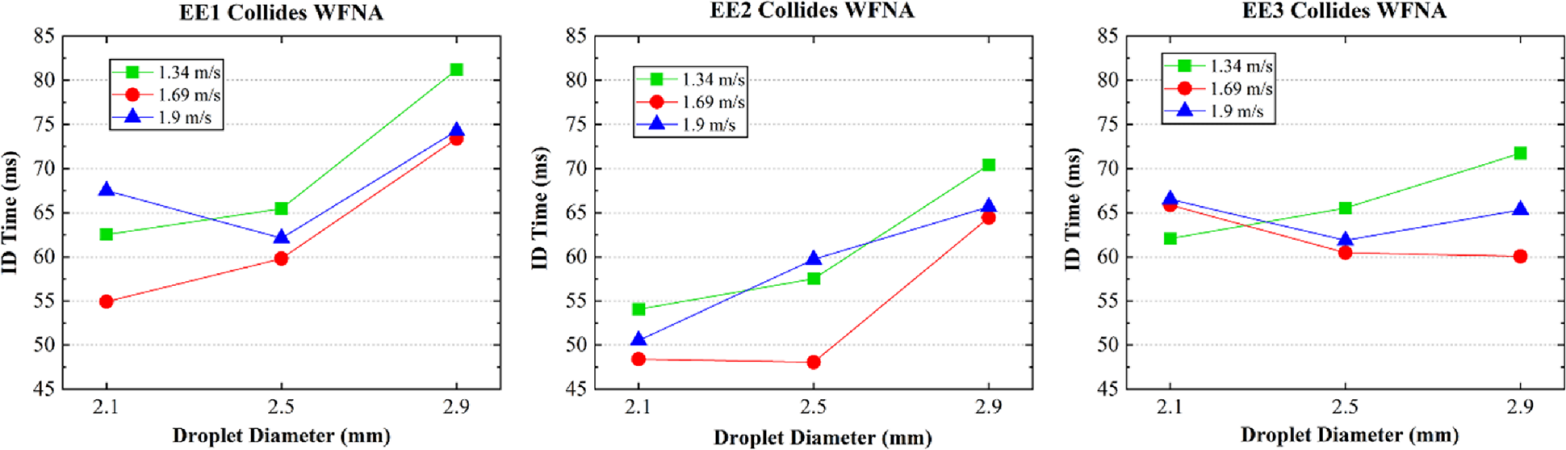
Statistics pertaining to the ignition delay time.

A strong hypergolicity generally signals an ability to react faster and to release more heat to rapidly increase a system temperature, which ultimately leads to a shorter ID time.^22^ The quiddity of hypergolic ignition is that the heat generation rate of the chemical reaction exceeds the heat dissipation rate of the system, i.e., the chemical reaction accelerates exponentially through the gas mixture heated by an excessive temperature. This process occurs repeatedly until fuels burn.^23^ In this study, the entire process was recorded using a camera, and the resting images are shown in Figure 3. Following the droplet (EE) colliding with the WFNA, these two intermiscible phases initially mixed in a hydrodynamic manner. Then, the gas mixture was generated via a chemical reaction, and an explosion occurred when the concentration of the EE or PE in the WFNA reached a certain value. According to the statistics pertaining to the ID time (Figure 4), the droplet size of EE1 and EE2 was anticipated to be small, and the velocity of the collision was expected to be 1.69 m/s. Overall, the EE2 mixture may be a suitable mixed-fuel option since its ID time was shorter compared with EE1.

**Figure 5:**
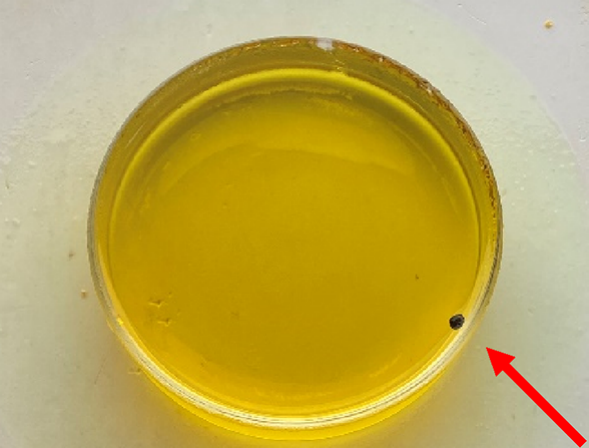
Black carbonaceous solid (indicated by the red arrow) in WFNA.

In the experiments, the large size of the droplet often resulted in two scenarios, i.e., the failure of hypergolic ignition or an excessive ID time, which caused a violent explosion. Figure 5 shows a failed case in which a black carbonaceous solid was generated via a chemical reaction in the absence of complete combustion; the chemical reaction occurred on the droplet’s surface before the two liquid phases had completely mixed because the concentration of EE in the WFNA was insufficient.

### 3.2 Simulation Results

**Figure 6:**
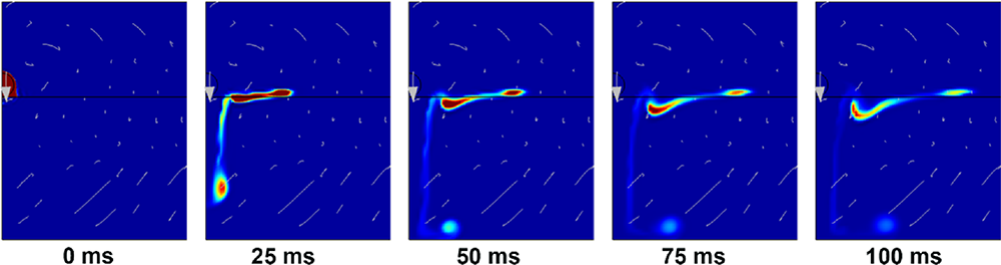
Computed results for the mixing process.

Figure 6 shows a two-dimensional axisymmetric model of the mixing process over 100 ms in the case where no chemical reaction occurred. The droplet of ethylene glycol with a φ of 2.5 collided with the liquid pool of HNO_3_ in 1.69 m/s. Following the collision, a mass of ethylene floated on the HNO_3_, whereas the remaining portion rushed to the bottom of the pool.

**Figure 7:**
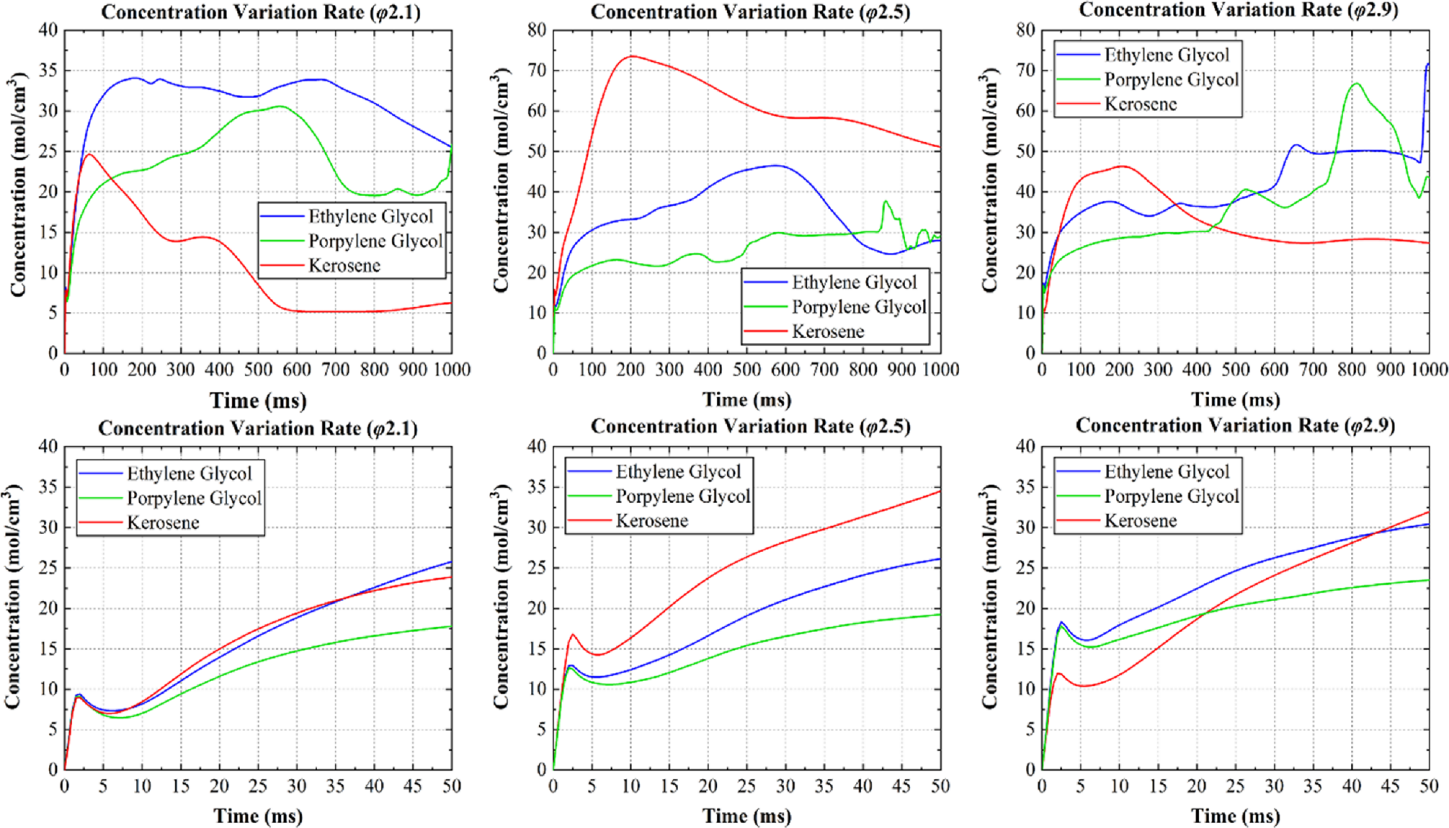
Changes in concentration of fuels in HNO_3_.

Figure 7 shows changes in the fuel concentration in the HNO_3_ process during the first second and the first 50 ms of the simulation, in which the collision velocity was 1.69 m/s. The performance in the first 50 ms was considerable because the ID time was expected to be less than 50 ms. The concentration of kerosene consistently underwent a sharp and rapid drop following substantial growth, which indicated a disappointing intermiscibility between kerosene and HNO_3_. Additionally, the alcohol fuels, particularly the ethylene glycol, exhibited a relatively stable concentration following an initial increase, with the ethylene glycol reflecting a higher concentration during a 1 s period and a higher speed from the start (50 ms) compared with the propylene glycol.

**Figure 8:**
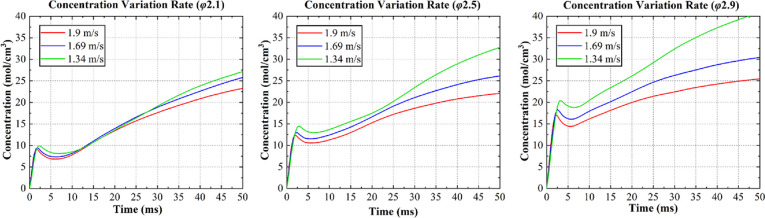
Changes in the concentration of ethylene glycol.

Given the stability and velocity of intermiscibility, ethylene glycol was determined as an applicable fuel, and further calculations using this fuel were performed (Figure 8). Here, considering the high-speed mixing from the start (50 ms), the mixing tendency of the ethylene glycol with the HNO_3_ indicated that the collision velocity could be expected to be slow when the droplet size was large and more rapid when the droplet was small.

### 3.3 Specific Impulse Calculation Results

**Figure 9:**
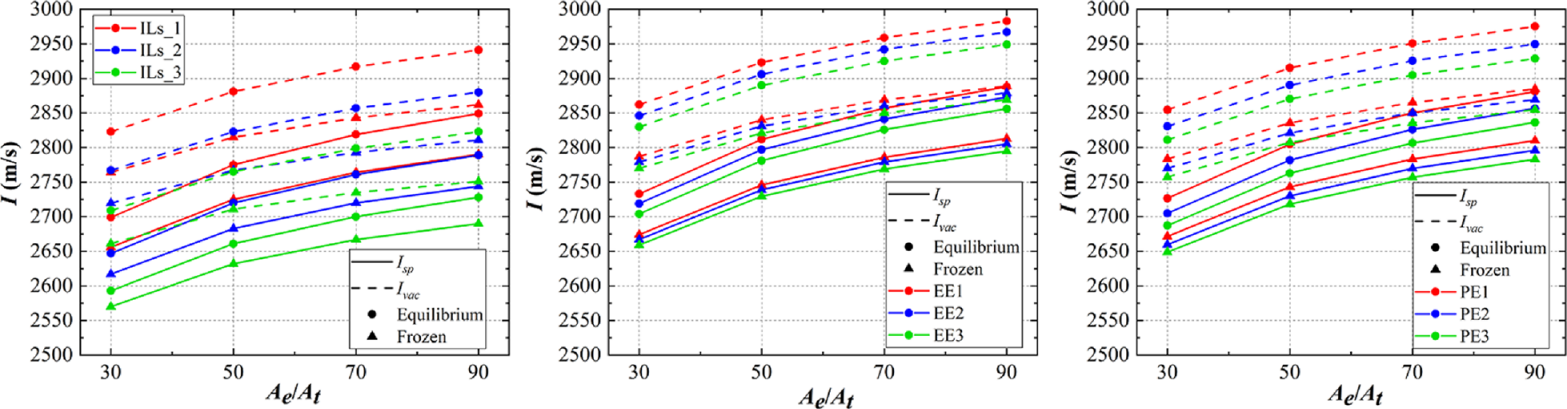
Specific impulse calculation results.

Figure 9 shows the results of specific impulse (*I*) calculation with every fuel. Specific impulse, on the whole, is increased in logarithmic growth with the nozzle area expansion ratio (*A*_e_/*A*_t_). The nozzle area expansion ratio depends on the structure of rocket engine, which is barely changed once decided. It makes more sense to compare in the same ratio. As for the specific impulse in vacuum (*I*_vac_), it is higher than in nonvacuum (*I*_sp_), and the results in equilibrium is higher than those in frozen, which are common sense. The specific impulse is increased with relative content of WFNA (oxidizer), in which the fuel that only contains [EMIM]DCA (ILs) is more sensitive to the change of content. In the same condition, EE (ILs mixed with ethylene glycol) gets the highest *I*_sp_ and *I*_vac_ compared to PE ((ILs mixed with ethylene glycol) and ILs. It is observed that mixing a small amount of alcohol fuels can increase the specific impulse.

**Figure 10:**
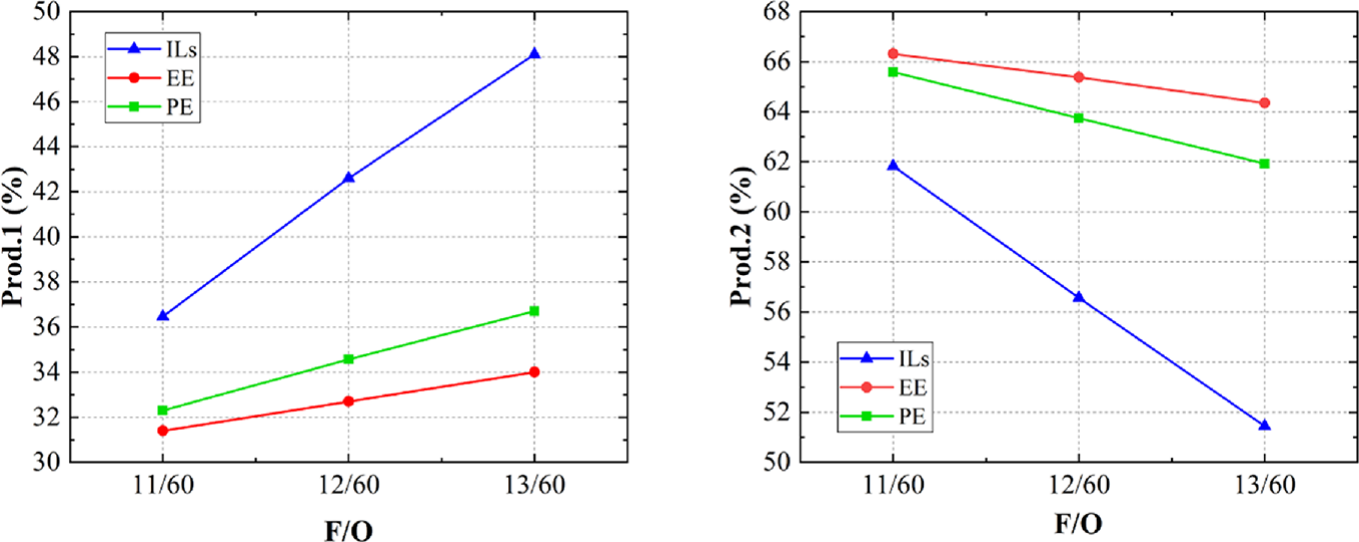
Proportions of combustion products of three fuels.

As for the products for the reaction of fuels (PE/EE/ILs) and oxidizer, Figure 10 shows the trends under different mole fraction ratios of fuels and oxidizers (F/O). The products were divided in two groups: Prod.1 group includes CO and H_2_, which were regarded as the products of incomplete combustion; Prod.2 group includes N_2_, O_2_, CO_2_, and H_2_O, which were regarded as the products of complete combustion. The sum of the proportions of these two groups is close to 100% (97–99.5%), and the rest are other particles. Prod.1 and Prod.2 are changing in a linear fashion if the variable is F/O. When the fuel is an IL, the products get more Prod.1 than PE and EE, which makes a lower specific impulse because of more incomplete combustion. The change of ILs’ Prod.1 or Prod.2 is much more sensitive to the relative content of WFNA (oxidizer), which is consistent with the calculation results of specific impulse above. EE and PE can cause more complete combustion, and EE causes the most complete one.

## 4 Discussion

In this work, the hypergolic ignition ID time and the behavior of EE mixed with WFNA were reported using high-speed photographic techniques. Furthermore, the mixing was calculated using the TPF method when ethylene glycol, propylene glycol, and kerosene were used as droplets and HNO_3_ as the liquid pool. At last, different fuels’ specific impulses were calculated by the CEA code.

The results demonstrated the feasibility of hypergolic ignition when mixing common fuels (particularly alcohol fuels) with [EMIm]DCA. The selection of alcohol fuels should follow the following principles: (i) the alcohol fuel and the ILs must be immiscible at any given proportion; (ii) the viscosity of the alcohol fuel should be as low as possible; (iii) the alcohol fuel must be both combustible and economical.

The results of the simulation experiment, which was used as a predictive method, were not entirely consistent with the experiment results, likely because the ignition entailed a chemical–hydrodynamic coupling model, but the simulation only considered the aspect of hydrodynamics. Despite this limitation, the simulation results could provide a reference for the selection of the parameters that are used in various engineering applications, with the results indicating that the collision velocity should be as high as possible and the droplet as small as possible. If the droplet is large, the collision velocity will be low, resulting in the failure of hypergolic ignition, which occurred during this study’s experiments.

Based on the analysis, WFNA can be expected to collide with atomizing EE at high speeds. However, additional phase-solubility analysis related to atomizing EE and WFNA can be conducted using the finite element method alongside supplementary parameters, including the angle and the velocity of the squirt, the atomizing level, and the content of the alcohol fuel, all of which can be calculated to provide further guidance for engineering applications.

Meanwhile, the thermodynamic computing backed up the feasibility of using blend fuels as a propellant by a theoretical method: the specific impulse of blend fuels is higher than ILs. For alcohol fuels ((CH_2_OH)_2_, C_3_H_8_O_2_), the energies of the C–C bond and C–H bond are high, the standard enthalpies of formation are low, but the combustion may be more complete because of atom of oxygen in it. That is the reason why specific impulse of EE is higher than PE: C_3_H_8_O_2_ has more C–C bonds and C–H bonds, which leads to the lower standard enthalpies of formation.

From a practical application point of view, the choice of propellant should take into all these factors: the performance of hydrodynamics and thermodynamics, toxicity, and economy.
